# Fracture Properties Evaluation of Cellulose Nanocrystals Cement Paste

**DOI:** 10.3390/ma13112507

**Published:** 2020-05-31

**Authors:** SeyedAli Ghahari, Lateef N. Assi, Ali Alsalman, Kürşat E. Alyamaç

**Affiliations:** 1 School of Civil Engineering, Purdue University, West Lafayette, IN 47907, USA; sghahari@purdue.edu; 2 Studies and Research Unit, Al-Mustaqbal University College, Babylon 51001, Iraq; 3 Department of Civil Engineering, University of Arkansas, Fayetteville, AR 72701, USA; alialsalman@email.uark.edu; 4 Tatum-Smith-Welcher Engineers Engineers, Rogers, AR 72758, USA; 5 Department of Civil Engineering, Faculty of Engineering, Firat University, Elazig 23200, Turkey; kealyamac@firat.edu.tr

**Keywords:** fracture, cellulose nanocrystals, renewable materials, sustainable infrastructure

## Abstract

Due to the need for high-performance and sustainable building materials, the investigation of the determination of fracture toughness of cement paste using new and sustainable materials, such as cellulose nanocrystals (CNCs) is worthwhile. Contrary to other well-known nano-reinforcement particles, such as carbon nanotubes, CNCs are less toxic; therefore, they have less safety and environmental risks. Fracture behavior of cement paste has been studied intensively for a long time. However, the incorporation of new materials in the cement paste, such as cellulose nanocrystal materials (CNCs), has not been fully investigated. In this paper, the fracture behavior, compressive strength, and hydration properties of cement paste reinforced with cellulose nanocrystal particles were studied. At the age of 3, 7, and 28 days, a three-point bending moment test, and a calorimetry and thermogravimetric analysis, scanning electron microscopy (SEM), and energy dispersive x-ray spectroscopy (EDX) analysis were performed on the water-to-binder-weight ratio of 0.35 cement paste, containing 0.0%, 0.2%, and 1.0% volume cellulose nanocrystals. Results indicated that the fracture properties and compressive strength were improved for the sample containing 0.2% CNCs. Preliminary results indicate that CNCs can improve the fracture behavior of cementitious materials and can be considered as a renewable and sustainable material in construction.

## 1. Introduction

Concrete structures experience a substantial number of loading/unloading cycles throughout their service lives. Therefore, the structures are designed with regard to potential fatigue loading distress. Fracture mechanics provides a broad vision of how the size of structural elements may affect the ultimate capacity of the entire structure. It is also an invaluable tool to predict crack propagation patterns. Accordingly, the fracture design of structures must be reviewed when new materials, such as fibers, carbon nanotubes, cellulose nanocrystals (CNCs) [1,2,3], etc. are introduced. Elastic modulus, tensile and flexural strength, fracture energy, and impact resistance have been proved to be enhanced by the incorporation of nano-reinforced materials. However, an environmental impact, health and safety issues, and cost-benefit analysis are yet to be considered as important factors when it comes to the usage of such materials [3]. 

CNCs are considered “green materials”, due to the fact that when substituted by cement particles, they cause a significant reduction in CO_2_ emission; CNCs are rod-like nanoparticles extracted from the plants and trees. They are less toxic compared with other nanoparticles and have less safety and environmental risks, since they are biodegradable and non-petroleum-based materials. Because of their high aspect ratio and elasticity, CNCs are suitable for several applications in cementitious materials. A significant liability of nanoparticles is that the surface needs to be functionalized in order to react with the host particles, i.e., cement clinker. However, CNCs have reactive surfaces, and more importantly, they are hydrophilic materials. All such promising properties make CNCs a possible alternative product for complementary cementitious materials. 

Fracture toughness is an essential property of concrete structures when evaluating the serviceability and estimating the life cycle needs to be addressed. Fracture toughness denotes the capacity of a specimen to resist fracture. Cementitious materials such as cement paste are known as quasi-brittle materials with low tensile strength and fracture toughness, which makes them susceptible to cracking. The potential application of many types of nanomaterials have been studied to produce sustainable cement-based materials, and the positive effect of such materials on the mechanical properties including fracture toughness of cement paste have been expressed in the literature. 

Several studies have been conducted to investigate the effect of fibers, CNCs, carbon nanotubes (CNTs), and polymers on Portland cement properties. Land and Stephen [4] examined the effect of incorporating C-S-H seeds on cement paste properties. They found that adding 0.5% C-S-H seeds by weight of the cement reduced the capillary porosity of the hardened cement paste due to the increase in hydration products and heat of hydration. The compressive strength was enhanced by 3% and 33% at 24 h and 28 days, respectively. Rocha et al. [5] investigated the influence of CNTs on the fracture properties of cement paste composites. They reported an increase in fracture energy of up to 90% by using 0.05% CNTs by the weight of cement compared to the reference paste (0% CNTs). Hu et al. [6] also reported enhancement in fracture energy and fracture toughness by the incorporation of 0.1% of CNTs by the weight of cement. The improvement was 43% and 19%, respectively, as compared with the reference paste. Liu et al. [7] found approximately the same behavior, with 0.5% CNTs by weight of cement. 

Silva and Monteiro [8] examined the effect of polymers, hydroxypropyl methylcellulose (HPMC), and poly acetate (EVA), on the hydration process. The results showed that HPMC enhanced inner product formations, while EVA inhibited ettringite crystal formations. Dousti et al. [9] incorporated CNCs by approximately 0.5% of cement paste and investigated the influence on mechanical properties. Both compressive strength and tensile strength were reported to be enhanced with the incorporation of CNCs. This enhancement was attributed to the increase of hydration at an early age. The increase at one day of age was significant: approximately 60% compared to the reference sample. The difference in strength was found to be 30% at the age of 56 days. On the other hand, Flores et al. [10] stated that the incorporation of CNCs delayed hydration at an early age, yet enhanced hydration at later ages. Additionally, with the incorporation of CNCs, high-density C-S-H was found in cement paste. Mejdoub et al. [11] studied the effect of nanofibrillated cellulose on the mechanical properties of cement paste. The results showed that there were significant improvements in compressive strength, porosity, and hydration product formation, by adding CNCs to the mixture. The compressive strength of cement paste was enhanced by 43%, using 0.3% nanofibrillated by weight of cement.

To the best of our knowledge, the fracture properties of cement paste containing cellulose nanocrystals has not been fully evaluated and studied yet. In this paper, the effect of CNCs on the fracture behavior, compressive strength, and hydration properties of cement paste was investigated. The fracture behavior of cement paste reinforced with CNCs was investigated at the age of 3, 7, and 28 days. A three-point bending moment test, and a calorimetry and thermogravimetric analysis, were performed on the binder matrix, with a 0.35 water/binder ratio. CNCs were added to the binder as a replacement for cement materials. The replacement ratios were set to be 0%, 0.2%, and 1.0% by volume of cement. A scanning electron microscope (SEM) (Gemini SEM/EDX—ZEISS, Baden-Württemberg, Germany) was utilized to investigate the effects of cellulose nanocrystals on microstructural properties of the paste. Preliminary results indicated that CNCs can improve the fracture behavior, compressive strength, and hydration properties of cementitious materials, and such materials can be considered as a renewable and sustainable material in construction.

## 2. Experimental Investigation

### 2.1. Materials and Mixing Method

Cement paste mixtures were prepared using the following main constituents: Portland cement type I 42.5 R (C), polycarboxylate ether-based superplasticizer (SP), and CNCs. The physical and chemical properties of CNCs and Portland cement are shown in Table 1, Table 2, and Table 3, respectively. CNCs were in the form of dispersed suspension (11.9 at. % CNCs in water). The cement pastes were prepared by using a vacuum mixer (high-shear mixer), to reduce the entrapped air voids in the mixes and reduce the potential agglomeration of CNCs.

The mixing procedure and specimens’ production included the following steps: (1) cement, CNCs suspension and water were mixed for 2 min, at 400 rpm; (2) walls of the mixer were scraped using a spatula, for 30 s; (3) the SP was added to the mix, and the mixing was done for 2 min; (4) Cement paste was poured into molds; (4) specimens were removed from the molds 24 h later; (5) samples were cured in an environmental chamber, at a constant temperature of 50 °C, until the day of the test.

After the completion of mixing, tests were immediately conducted to determine mini-slump flow diameter, according to ASTM C1437 [12]. For the slump flow test, a truncated conic mold was placed on a smooth plate, filled with the cement paste, and lifted upward. The spread diameter of the mortar was measured in two perpendicular directions and the average of the diameters was reported as the slump flow deformation of the paste. Three series of cement pastes were considered for this study. As shown in Table 4, for all of the mixes, the water-to-cement ratio was fixed at 0.35, to create a reasonable comparison. The authors selected high and low concentrations of CNCs to generate a noticeable effect of incorporations [11]. 

### 2.2. Compressive Strength Test

Four prisms were tested for each percentage of CNC incorporation, to evaluate the compressive strength of the paste. The dimensions were measured 38.1 mm × 38.1 mm × 152.4 mm (1.5 in × 1.5 in × 6 in). The dimensions were selected so as to ensure a tension failure of uniaxial compression load and to examine the effect of CNC percentage on the compressive strength of the cement pastes. The specimens were cast vertically, to obtain a smooth surface, and vibrated for 10 s. The compressive strength was evaluated at 28 days.

### 2.3. Fracture Test Setup

To study the fracture behavior of cement paste, three-point bending beam specimens were prepared. Several different notch/beam-length ratios have been proposed for this test: The International Union of Laboratories and Experts in Construction Materials, Systems and Structures (RILEM) suggests a ratio of 0.5 for notched beams [13]. Elices and Planas considered a ratio between 0.2 to 0.5 as a practical experimental range [14]. It is suggested by Jeng and Shah [15] that for determining fracture energy, specimens must have a central notch with a depth size equal to half of the beam depth + 5 mm; and the width of the notch should be less than 5 mm. Moreover, the initial notch/depth ratio must be equal to (1/3). This notch size was also used by Gettu et al., in a study to find the specimen size effect on the fracture behavior of high strength concrete [16]. 

The notch should be sawn under wet conditions. However, if the notch is cast, during demolding, the beam must be taken out with extra care. For each mixture design, at least four specimens are required to be tested. It has been suggested to cut the notch before the drying of the sample, to avoid the formation of fine cracks during the cutting process.

In this study, ASTM C1421 standard [17] was followed. The ratio of the span to the depth of the beam (S/D) and the ratio of the notch length to the beam depth (a_0_/D) were 3 and 0.33, respectively. The thickness of the beams was equal to the beam depth 25.4 mm (1 in). Thin blades with a thickness of 0.2 mm were fixed to the molds during the curing process to make the notches. The fracture test was performed using a Measure Test Simulate machine (MTS300, MTS Systems Corporation, Eden Prairie, MN, USA). The load rate was kept at 0.1 mm/min for all notched beams, as recommended by ASTM C1421 [17].

### 2.4. Hydration Process

In order to study the hydration process of cement paste, the heat flow rate and cumulative heat release were measured with a TAM Air isothermal calorimeter (TAM Air 3-channel, TA Instruments, New Castle, DE, USA). Before the test, TAM was set to stabilize the baseline for 30 min. After 10 min of mixing, the cement paste was poured into the ampoules. Sealed ampoules were then inserted into the TAM chamber. The device started to measure the temperature after 45 min, to reach the required equilibrium [18].

## 3. Experimental Results and Discussion

### 3.1. Compressive Strength 

**Figure 1:**
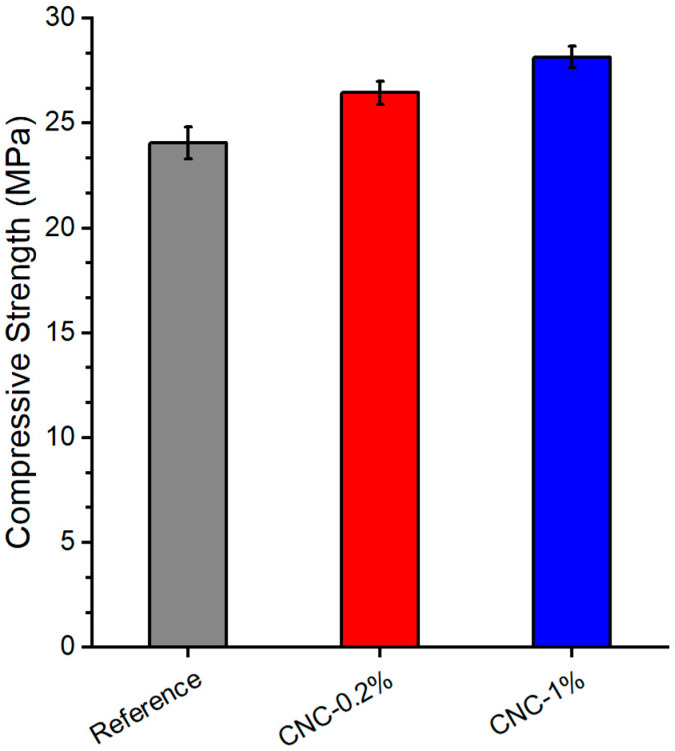
Effect of CNCs % on compressive strength.

Figure 1 presents the effect of CNC % on the compressive strength of the cement paste specimens. As the CNCs volume increases, compressive strength increases. The incorporation of 0.2% and 1% CNC increases the strength by 10% and 17%, respectively. The increase in the compressive strength could be attributed to the hydrophilic characteristics of CNCs, which results in higher hydration products. Additionally, the high specific surface area of CNCs enhances the paste–CNCs interface. This leads to the enhancement of stress transfer between cement paste and CNCs, and consequently increases the compressive strength [9,10]. Furthermore, the use of 0.2% and 1% CNC reduces the volume of cement, while the compressive strength is not compromised. Therefore, a sustainable and eco-friendly paste matrix can be produced. It is worthwhile noting that, although there is an increase in the compressive strength for 1% CNC, the fracture properties are affected. 

### 3.2. Fracture Analysis 

Several experimental studies have been carried out to study the effect of multiple parameters on critical stress intensity factor (*K_c_*) and critical energy release rate (*G_c_*). Experimental investigations show that the fracture toughness of concrete increases with the increase of aggregate volume, maximum-size aggregate, and roughness of the aggregate particles. On the other hand, roughness decreases with increasing water/binder ratio and increasing air content [19]. The crack path in concrete depends on the mechanical interaction between aggregates and the binder paste matrix [20]. Furthermore, the fracture energy of concrete is dependent on the deviation of the crack path from an idealized crack plane [21]. It is well-known that cracks occur when a material reaches to its ultimate tensile strength. Microcracks already existent in the interfacial transition zone (ITZ) between binder matrix and coarse aggregate, before the application of an external load, due to the difference in the elastic modulus of cement components. The microcracks, during a fatigue cyclic loading, grow and reach each other, which is called a crack propagation (about 75% of the ultimate load), and after the first loading cycle, the structure fails [22].

In general, cracks propagate perpendicular to the maximum tensile strength and through the weakest path. The simpler a structure’s shape is, and the close its stress intensity factor is to 1, the faster the cracks propagate. Theoretically, the stress strain curve for an ideally brittle material, which reacts in accordance with linear elastic fracture mechanics (LEFM), is linear until the maximum stress peak point. On the contrary, the non-linear curve is observable in quasi-brittle materials; before the peak of the curve, micro cracks begin to propagate and reach each other; at the peak stress, the localized macrocracks lead to the failure of the structure. In the pre-peak stage, “strain-softening” occurs due to steady-state crack propagation [22,23]. 

**Figure 2:**
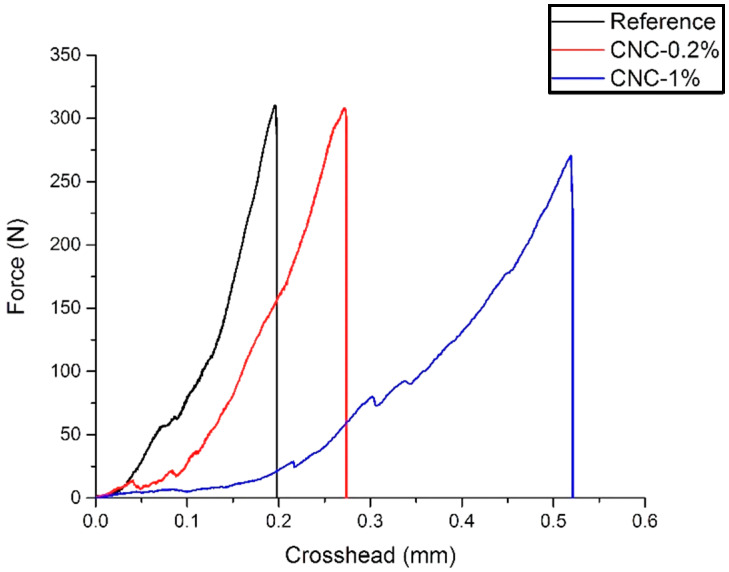
Three-point bending test for the three days samples.

Fracture toughness denotes the ability of a specimen to resist fracture. A typical experiment that identifies the fracture properties of the materials is a three-point bending moment test [24]. The results of three-point bending test for the three days specimens are presented in Figure 2. The addition of 0.2% CNCs shows approximately the same peak load as the reference mixture. However, the incorporation of 1% CNC leads to a decrease in the peak load. This is probably due to the agglomeration of CNCs. Additionally, for the CNC-1% sample, the rate of load increase is less than the reference and the CNC-0.2% samples. This might be due to the inconsistency of the ‘notched specimens’ crack size. For all CNC-1% samples, it is observed that the crack width is wider than 0.2 mm. This might be due to time-dependent deformation, such as shrinkage, which occurs in the sample with the highest concentration of CNCs. 

**Figure 3:**
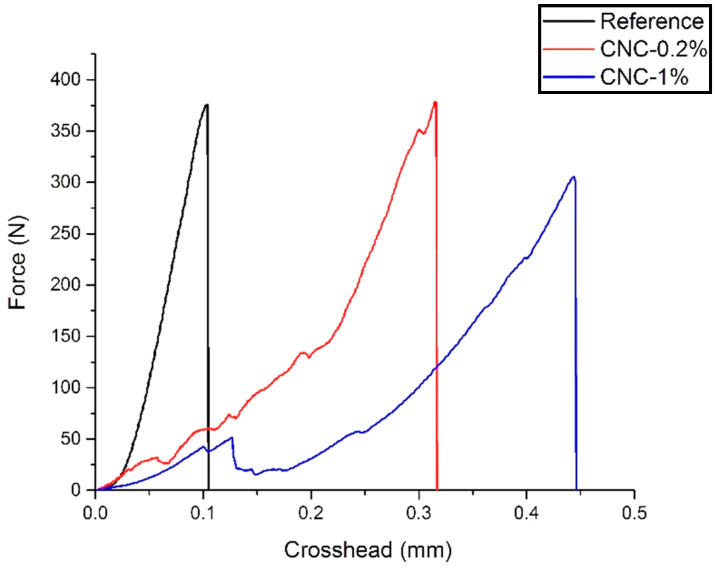
Three-point bending test for the seven days samples.

**Figure 4:**
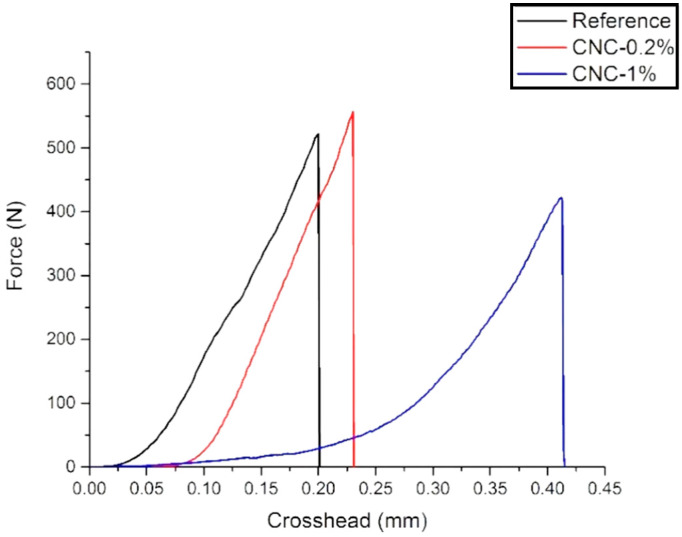
Three-point bending test for the 28 days samples.

Figure 3 shows the seven days results. No significant change can be observed compared to the three days samples. CNC-1% gives the lowest peak load among all samples. Additionally, the trend of the load rate increase is still lower than that of the other two samples. The results of the three-point flexure test for 28 days are presented in Figure 4. As is shown, the addition of 0.2% CNCs leads to an increase in the peak load. This enhancement can be attributed to the increase of the C-S-H formation, causing a higher modulus of elasticity.

**Figure 5:**
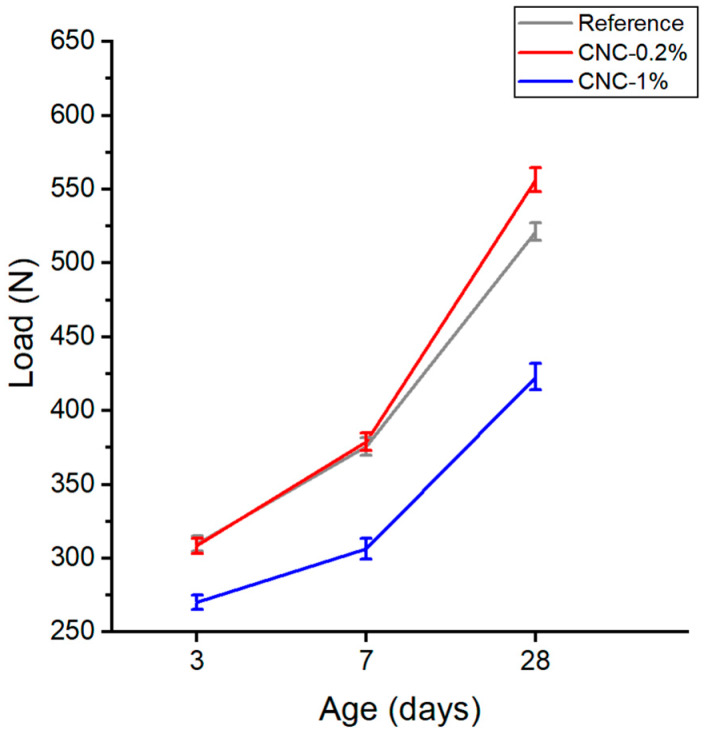
Average of the peak load at 3, 7, and 28 days.

The average of the peak load for four samples, along with the standard deviation error bars, are given in Figure 5. The results indicate that the addition of 0.2% CNC shows almost the same value as the reference. However, a significant improvement can be observed at 28 days. The addition of 1% CNC leads to a decrease in the peak load, regardless of the age of the specimens. There are two possibilities to explain this behavior; large crack width might cause the notched beam sample to fail at a lower peak load. Additionally, the inefficiency of the addition of CNCs to improve the fracture behavior might be due to the improper dispersion of CNCs in the mixture. Due to their high surface energy, CNCs have a distinct propensity towards agglomeration. The dispersion of nanoparticles within the cement paste is a significant factor leading to the performance of such products. When nanoparticles are added in excess to the mixture, CNCs are not uniformly dispersed in the cement paste, and consequently, weak areas appear in the cement mortar. Therefore, the dispersion of nanoparticles is crucial to achieving composite materials with improved properties [11]. It is worthwhile to note that, due to the different force fields and attraction forces of cement to cement particles, and CNCs to CNCs, CNCs should be dispersed well in the mixture. Otherwise, the expected improvement after adding CNCs will not be achieved.

The incorporation of CNCs by 0.2% by volume of cement, enhances the properties and microstructure of cement paste. However, when a high concentration of CNCs is used in the mixture, a decrease in the strength of the cement paste is recorded. This could be caused by CNCs agglomeration. If the agglomeration of CNCs is minimized, the additional enhancement of cement paste strength might be obtained. This can be resolved by using a high shear mixer to separate the particles. The other approach is using ultrasonication to break the agglomerates.

Fracture toughness was calculated according to the ASTM C1421 [17] using Equation (1)

(1) $$k_{lpb}=g\left[\frac{P_{max}S_{0}10^{-6}}{BW^{3/2}}\right]\left[\frac{3{\left[a/W\right]}^{1/2}}{2{\left[1-a/W\right]}^{3/2}}\right]\;g=g\left(a/W\right)\;=\frac{1.99-\left[a/W\right]\left[1-a/W\right]\left[2.15-3.93\left[a/W\right]+2.7{\left[a/W\right]}^{2}\right]}{1+2\left[a/W\right]}$$

where *k_Ipb_* is fracture toughness (MPa $\sqrt{m}$) for a single-edge pre-cracked beam, *g* is a function of the ratio *a/W* for three-point flexure, *P_max_* is maximum force (N), *S*_0_ and *S_l_* are outer and inner span (m), respectively, *B* is side to side dimension of the test specimen perpendicular to the crack length (depth), *W* is top to the bottom dimension of the test specimen parallel to the crack length (depth), and a is crack length (m). 

**Figure 6:**
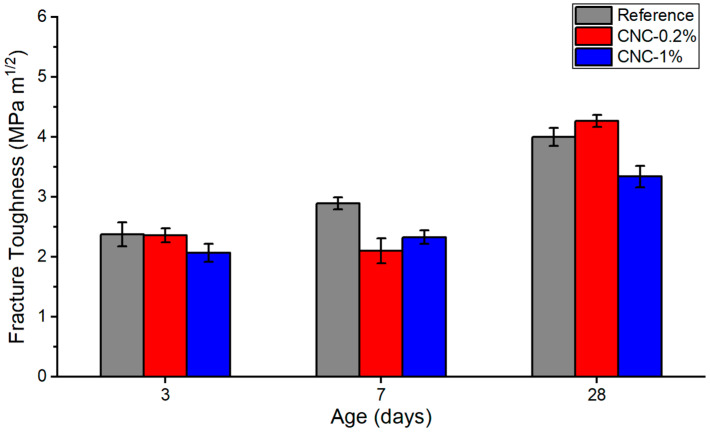
Fracture toughness at 3, 7, and 28 days.

**Figure 7:**
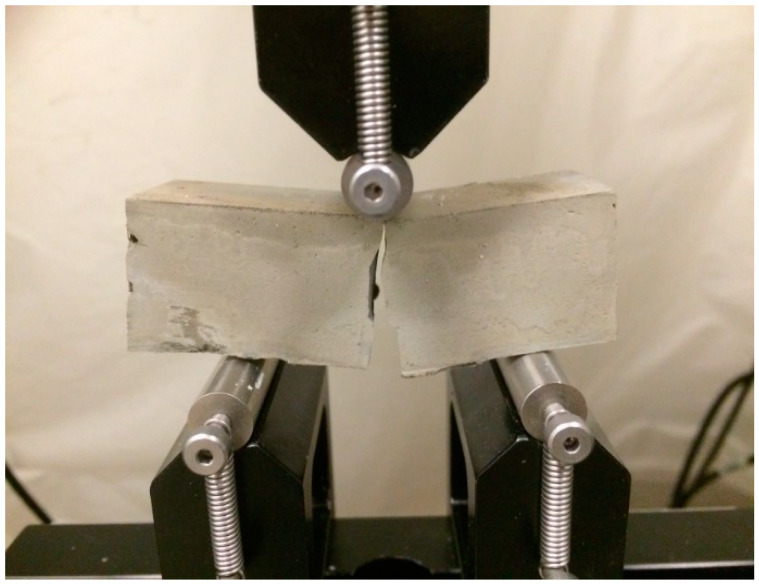
The propagation of the crack during the three-point bending test.

The calculated fracture toughness versus three different ages is presented in Figure 6. As expected, the CNC-0.2% samples give the highest value among all samples. This is attributed to the increase in the flexural strength of concrete containing CNCs, which is an indirect method to measure the tensile strength of concrete. On the other hand, the addition of 1% CNCs leads to a decrease in the fracture toughness, due to the agglomeration of CNCs at higher concentrations that act as stress concentrators (i.e., defects) in the cement paste. Additionally, the standard deviation of CNC-1% samples has the highest value among all samples. Figure 7 shows the propagation of the crack during the three-point bending test for CNC-1%. The picture is taken at the moment of the failure, which shows a significant bending, regarding the fact that the specimen is brittle; also, as can be seen from the trend that CNC-1% follows at all ages, such nanoparticles could have made the samples more ductile. This fact indicates that the cellulose nanocrystals could have the bridging effect due to their high aspect ratio; CNCs are believed to be able to bridge microcracks and that such nanomaterials might act similarly to what is expected from steel or polypropylene fibers [1]. 

### 3.3. Hydration Analysis

**Figure 8:**
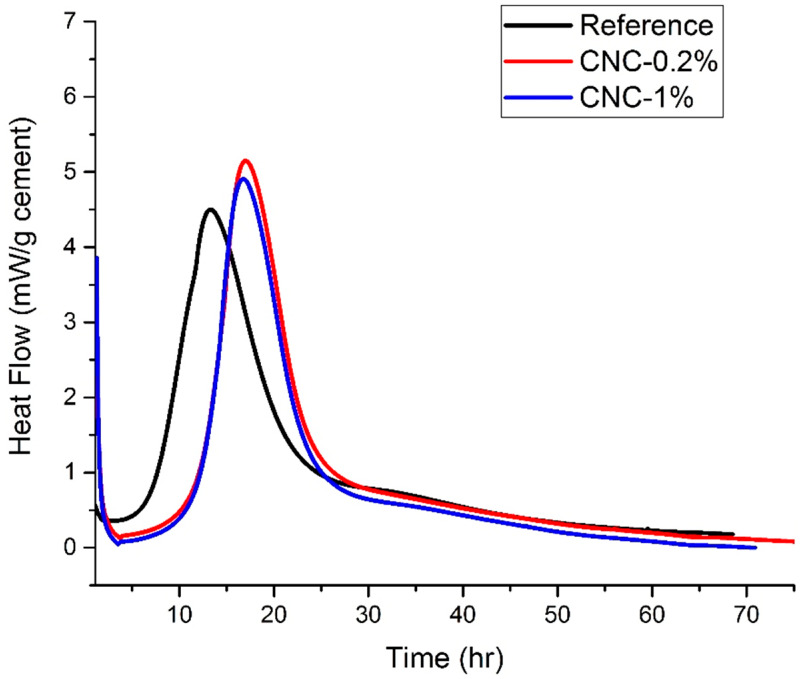
The heat flow of all samples versus time.

The results of the heat flow for all samples are presented in Figure 8. The addition of CNCs leads to an enlargement in the dormant period, which results in a delay in the hydration reaction. The results indicate that the heat flow of the reference sample reaches at 13 h, while the peak is reached at around 16 h for the mixture with 0.2% CNCs and 1% CNCs. This could be due to the fact that CNCs adhere to the cement particles and reduce the reaction surface area between cement and water, and consequently, hydration slows down—which is similar to the role of high range water reducer admixtures. Moreover, this retardation effect might be due to the deposition of CNCs on the surface of three calcium silicate (C_3_S) phases of cement particles. The latter can form a thin layer, which acts as a barrier layer and blocks the cement particles from being hydrated further. In general, the incorporation of CNCs results in a higher heat flow compared to the reference samples. However, the specific consequences on the mechanical properties still need to be investigated.

### 3.4. Scanning Electron Microscopy (SEM) and Energy Dispersive X-ray Spectroscopy (EDX) Observations

**Figure 9:**
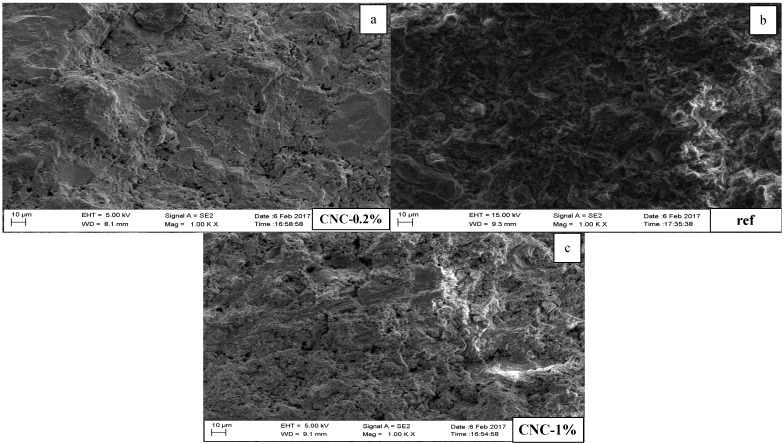
SEM images with 1000 resolution. (**a**) CNC-0.2% (**b**) reference sample (**c**) CNC-1%.

**Figure 10:**
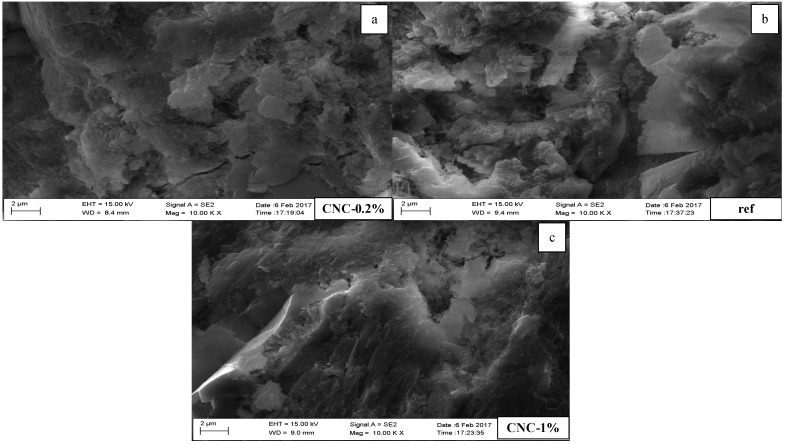
SEM images with 10,000 resolution. (**a**) CNC-0.2% (**b**) reference sample (**c**) CNC-1%.

The SEM test was conducted at 28 days. The cement paste samples were kept in an ambient condition until the SEM observations were performed. There are two magnifications, at 1000 and 10,000. Figure 9 illustrates three images with 1000 resolution for the reference sample, CNC-0.2%, and CNC-1.0%. The images reveal that the reference sample has more unreacted cement particles in comparison with the other specimens containing CNCs. CNC-0.2% and CNC-1.0% are free of microcracks, and the ITZs are smoother than the reference sample. Figure 10 shows differences in the microstructure formations. The reference sample contains unhydrated cement particle packets. CNC-1.0% has microcracks in comparison with CNC-0.2%.

In conclusion, the SEM observations have shown that the presence of CNCs enhances the hydration process of Portland cement in comparison with the reference sample. The samples incorporating CNCs look to be free of unhydrated cement particles, and the hydration products cover the surface of the samples. Furthermore, an increase in the ratio of CNCs may help to reduce the formation of microcracks, by bridging two sides of potential microcracks.

**Figure 11:**
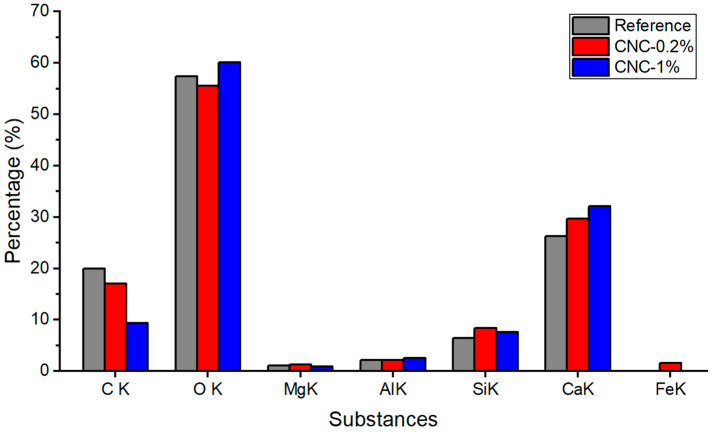
Energy dispersive X-ray spectroscopy (EDX) observations.

Figure 11 shows the energy dispersive X-ray spectroscopy (EDX)(Gemini SEM/EDX—ZEISS, Baden-Württemberg, Germany) observations for three representative locations for each sample, to get a better view of the material composition. The observation shows that for the samples with CNCs, the calcium and silicon product concentrations were increased. For instance, the concentration of calcium products is 28%, 26%, and 22% for CNC-1%, CNC-0.2%, and reference samples, respectively, while silicon product concentration is 7%, 7%, and 5%, approximately. The aluminum percentage is 2.50, 1.82, and 1.80 for CNC-1%, CNC-0.2%, and reference samples, respectively. The EDX observation proves that CNCs enhance the hydration process, leading to more hydration products, including C_3_A, C_3_S, and C_2_S.

## 4. Conclusions

This study aimed to evaluate the effect of cellulose nanocrystals (CNCs) on the fracture behavior, mechanical strength, and hydration of cement paste. An experimental program was conducted, using a three-point bending test setup. At the age of 3, 7, and 28 days, a three-point bending moment test, and a calorimetry and thermogravimetric analysis, were performed on the water to binder weight ratio of 0.35 cement paste, with 0.0, 0.2, and 1.0% volume CNCs. The future work on this topic could be directed to the investigation of other CNCs replacement ratios. The conclusions are as follows:

1. The incorporation of 0.2% and 1% CNCs increased the compressive strength by 10% and 17%, respectively.
2. The addition of 0.2% CNCs led to an increase in the peak load; however, the incorporation of 1% CNC resulted in a decrease in the peak load.
3. The dispersion of CNCs particles was found to be a critical factor that can significantly affect the mechanical properties.
4. The addition of CNCs led to an extended dormant period, which resulted in a delay in the hydration reaction. However, the addition of CNCs led to a higher heat flow, compared to the reference sample.
5. SEM and EDX results showed an enhancement in cement hydration products for 0.2% CNCs and 1% CNCs, as compared to the reference cement paste.

## Figures and Tables

**Table 1:** Physical and chemical properties of cellulose nanocrystals (CNCs).

Length (µm)	Width (nm)	ρ (g/cm^3^)	σ_f_ (GPa)	Cross-Section
0.05–0.5	3–5	1.6	7.5–7.7	Square

**Table 2:** Portland cement chemical compositions.

Items	Limit	Results
SiO_2_ (%)	-	19.7
Al_2_O_3_ (%)	6.0 max	4.5
Fe_2_O_3_ (%)	6.0 max	3.5
CaO (%)	-	63.1
MgO (%)	6.0 max	1.4
SO_3_ (%)	3.0 max	2.9
Loss on Ignition (%)	3.5 max	1.2
Insoluble Residue (%)	1.5 max	0.3
CO_2_ (%)	-	0.5
CaCO_3_ in limestone (%)	70 min	90

**Table 3:** Portland cement potential phase compositions.

Potential Phase Compositions	Limit	Results
C_3_S (%)	-	60
C_2_S (%)	-	11
C_3_A (%)	8 max	6
C_3_AF (%)	-	11
C_3_S + 4.7C_3_A (%)	100 max	90

**Table 4:** Mixture design for the reference sample and samples containing CNCs (kg/m^3^).

Material	Reference	CNC-0.2%	CNC-1%
Cement	589.4	588.2	583.5
Water	206.3	206.3	206.3
Superplasticizer	7	7	7
CNC	0	0.6	3

## List of Acronyms and Notations

a_0_/D	Notch length/depth
CNCs	Cellulose nanocrystals
CNT	Carbon nanotube
C-S-H	Calcium silicate hydrates
EDX	Energy dispersive X-Ray spectroscopy
EVA	Poly acetate
*G_c_*	Critical energy release rate
HPMC	Hydroxypropyl methylcellulose
ITZ	Interfacial transition zone (ITZ)
*K_c_*	Critical stress intensity factor
LEFM	Linear elastic fracture mechanics
S/D	Span/depth ratio
SEM	Scanning electron microscope
SP	Superplasticizer
